# Investigation on Strain Hardening and Failure in Notched Tension Specimens of Cold Rolled Ti6Al4V Titanium Alloy

**DOI:** 10.3390/ma15103429

**Published:** 2022-05-11

**Authors:** Hao Zhang, Tao Gao, Jian Chen, Xunpeng Li, Haipeng Song, Ganyun Huang

**Affiliations:** 1 School of Mechanical Engineering, Yangzhou University, Yangzhou 225127, China; mx120200511@yzu.edu.cn (T.G.); chenjian.tud@hotmail.com (J.C.); mz120190633@yzu.edu.cn (X.L.); 2 Sino-European Institute of Aviation Engineering, Civil Aviation University of China, Tianjin 300300, China; hpsong@cauc.edu.cn; 3 Tianjin Key Laboratory of Modern Engineering Mechanics, School of Mechanical Engineering, Tianjin University, Tianjin 300072, China; gyhuang@tju.edu.cn

**Keywords:** titanium alloy, Ti6Al4V, strain hardening behavior, strain localization, failure

## Abstract

Uniaxial and notched tension samples are utilized to investigate the damage and failure of titanium alloy Ti6Al4V. The strain fields on the samples are obtained by the digital image correlation (DIC) method. Strain localization occurs before fracturing in all samples, and the width and size of the localized zone are characterized. Slant fractures are observed in uniaxial and notched tension specimen, which indicate that the initiation and propagation of cracks in thin sheet specimens are highly affected by the shear stress. Numerical simulations were performed for identification of hybrid hardening laws, and the results were compared with the experiments. The influence of the stress triaxiality on damage mechanism of Ti6Al4V was analyzed by observation of the specimen fracture surfaces using SEM. The results show that a higher stress triaxiality facilitates the formation and growth of micro-voids, which leads to a decrement of strain at failure.

## 1. Introduction

Titanium alloys and especially Ti6Al4V are widely used in aerospace, ship-building, and biomedical engineering for their high strength, excellent mechanical properties, and good biocompatibility [1,2,3]. Ti6Al4V consists of a hierarchical microstructure with the predominant α-phase (hexagonal close packed, HCP) and β-phase (body centered cubic, BCC) dispersed in the grain boundary. The mechanical behavior of Ti6Al4V is quite complex due to the interaction between the crystallographic slip and the twinning of HCP microstructure [4]. In practical engineering applications, the material can usually be subjected to various loading conditions, including temperature, strain rate, loading paths, and loading repetitions [5,6]. It is important to investigate the failure mechanism under different loading conditions in order to accurately predict the potential failure of titanium alloy.

The damage and fracture behaviors of ductile material are strongly influenced by the stress state [7,8]. For example, during tension loading condition the ductile damage is mainly caused by the formation and growth of voids, whereas under compression and shear loadings, the dominant damage mechanism is the nucleation and growth of shear cracks [9]. The Lode angle parameter and the stress triaxiality are two key factors that are commonly used to evaluate the influence of the stress state on ductile damage [10]. The Lode parameter is associated to the third invariant of the deviatoric stress tensor, while the triaxiality is the ratio of the hydrostatic stress to the von Mises stress. The different stress states within a given material can be achieved experimentally by the specimen geometry and the applied loading conditions. Smooth round bar and flat dog bone-shaped specimens, which provide a homogeneous stress state in the gage section, are usually used to obtain the elastic and plastic material parameters [11]. The notched specimens, which have a different notch radius or notches in thickness direction (plane strain specimen), are used to provide a tension dominated stress with a stress triaxiality of more than 1/3 [12,13]. To study fracture under a shear dominated stress state with zero stress triaxiality, different specimens and corresponding devices have been developed, such as in-plane shear specimens [14,15], a circular grooved torsion test [16], and a butterfly specimen [17] under different angles and loading conditions. Hollow cylindrical samples show possibilities for investigating the stress dependent damage and fracture by tension-internal pressure or tension-torsion combining loadings [18]. The cruciform-like specimens have been used to study the plastic deformation and damage process of the material under biaxial stress states [19,20]. Gerke et al. [21] presented experiments of the biaxial cruciform X0-specimen to analyze the influence of the stress triaxiality and non-proportional loading on the damage behavior of sheet metals.

Furthermore, material models have been developed to reflect the plastic and hardening behavior of different materials and to facilitate corresponding numerical simulations. The combined experimental/numerical inverse method was developed to extend the yield curve by adjusting the characteristic parameters of the material hardening laws [22]. The Hollomon and Voce hardening laws were used to describe the transition of the strain hardening behavior from the power-law to the saturation type at low and high temperatures of dual-phase steels [23]. The flow behavior of titanium alloy under hot working conditions was investigated using an extrapolated model that was based on the Linear and Ludwick hardening laws [24]. A practical approach to extend typical stress—strain curves and obtain the mechanical properties of material is the digital image correlation (DIC) method, which has been effectively applied in acquiring the deformation field on the surface of the material and the structure [25,26,27,28]. 3D deformation measurement data and finite element (FE) analysis of expansion tests were utilized to determine the parameters of the Swift and Hollomon hardening laws of aerospace alloys [29]. An alternative hardening model, including the Swift and Voce hardening laws, was proposed to quantify the post-necking hardening phenomena in ductile materials [30]. The strain hardening parameters in thick high strength steel were inversely identified through DIC full-field strain measurements and FE model update technique [31]. However, the hardening behavior and intrinsic mechanisms in sheet metals are still unclear, and the relationship between fracture characteristics and stress state is also an ongoing discussion. There is a need for characterizing the deformation and fracture behavior if we are to better understand the strain hardening and damage mechanisms in titanium alloy.

In this work, uniaxial and notched tension tests are carried out to investigate the plastic deformation behavior and fracture mechanisms of Ti6Al4V under different stress states. The evolution of deformation fields on the sample during loading is analyzed by DIC. Furthermore, the fracture surfaces of specimens are analyzed to understand the failure characteristics for Ti6Al4V. An identification strategy is proposed to obtain the appropriate hardening model and related parameters by comparing the various hardening laws. The relationship between the stress state and damage behavior of Ti6Al4V is discussed.

## 2. Materials and Methods

### 2.1. Experimental Specimen and Setup

**Figure 1:**
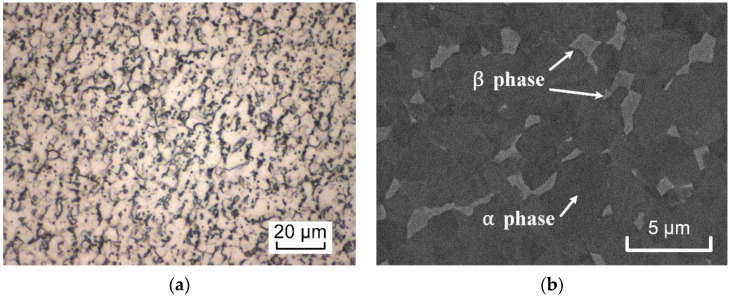
(**a**) Optical microscope image for the as-received Ti6Al4V; (**b**) SEM backscattered electron image showing α and β phases.

It is well known that the microstructure is a determinant for the mechanical behavior of titanium alloys [2,32]. Ti6Al4V is a typical dual-phase titanium alloy with HCP-structured α-phase and BCC-structured β phase. The HCP structure of α phase has fewer symmetric slip systems leading to the plastic anisotropy, whereas the BCC structure of β phase contents more slip systems. The as-received Ti6Al4V in this study is manufactured by a cold rolling process, and the thickness of sheet is 0.6 mm. The chemical composition of the as-received Ti6Al4V consists of 5.8% Al, 4.3% V, 0.15% Fe, and 0.0.2% C (on a mass basis), with the remainder consisting of Ti. Aluminum with a content of 5.8% is the stabilizer of α phase, and vanadium (4.3%) is the β-stabilizer. Figure 1 displays the microstructure and phase distribution of Ti6Al4V. It can be seen that the β phase has a small fraction and is distributed diffusely in Figure 1b.

**Figure 2:**
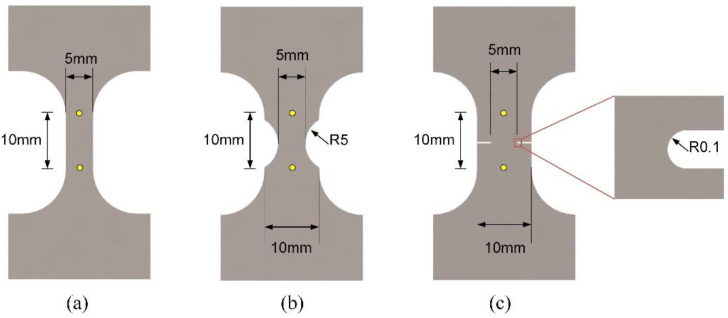
Sample geometry used for: (**a**) Uniaxial; (**b**) notched tension (R = 5 mm); (**c**) notched (R = 0.1 mm). The yellow points indicate the location for measuring relative displacement by digital image correlation (DIC).

The uniaxial and notched tension specimens are fabricated by a wire electrical discharge machine (GF AgieCharmilles, Schaffhausen, Switzerland); the size and dimensions of the test specimens are illustrated in Figure 2. The stress state of specimens can be characterized by the stress triaxiality and Lode angle relative parameters. The stress triaxiality η is used to characterize the stress state of specimens which is defined as

(1) $$\eta =\frac{\sigma_{m}}{\sigma_{eq}}$$

Here $\sigma_{m}$ is the mean stress, which is also called the hydrostatic stress, and $\sigma_{eq}$ the von Mises equivalent stress, which expressed as follows:

(2) $$\sigma_{m}=\frac{1}{3}I_{1}=\frac{1}{3}\left(\sigma_{1}+\sigma_{2}+\sigma_{3}\right)$$

(3) $$\sigma_{eq}=\sqrt{3J_{2}}=\sqrt{\frac{1}{2}\left[{\left(\sigma_{1}-\sigma_{2}\right)}^{2}+{\left(\sigma_{2}-\sigma_{3}\right)}^{2}+{\left(\sigma_{3}-\sigma_{1}\right)}^{2}\right]}$$

where $I_{1}$ and $J_{2}$ are the first invariant of the stress tensor and the second deviatoric stress invariant; $\sigma_{1}$,$\sigma_{2}$,$\sigma_{3}$ are the principal stresses following that $\sigma_{1}\geq \sigma_{2}\geq \sigma_{3}$.

**Figure 3:**
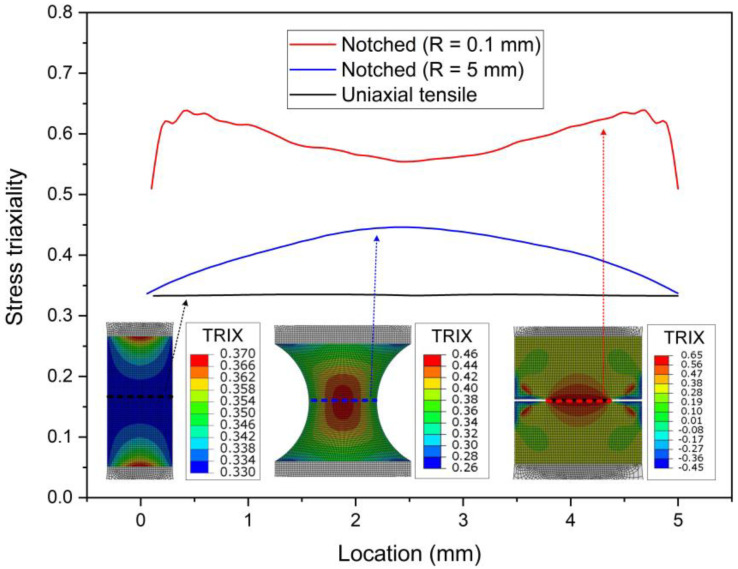
The initial stress triaxiality for the uniaxial tensile and the notched specimens. TRIX represents stress triaxiality.

The specimens are modeled by the FE program Abaqus/explicit (Abaqus 6.14, Dassault Systemes Simulia Corp., Providence, RI, USA). Five solid elements of 8-node reduced integration solid elements (C3D8R) are used along the thickness of the specimen. For all samples, the element size is 0.12 mm in the central gauge section, and it increases gradually to 0.15 mm for the transition region. The Young’s modulus 124 GPa and Poisson ratio 0.33 were used according to the uniaxial tensile tests. The initial stress triaxiality η in the central gage region is about 0.33 in the uniaxial tensile sample, while the initial stress triaxialities at the center are 0.45 and 0.58 in the notched tension samples (R = 5, 0.1 mm), as shown in Figure 3.

### 2.2. Experimental Procedure

The test specimens were loaded on a Deben Microtest 5 kN tensile stage device (Deben UK Ltd., Suffolk, UK), and a minimum of three samples for each loading condition were tested. The samples are subjected to a tensile deformation; the speed of the cross head is 0.6 mm/min for uniaxial tensile tests, and 0.3 mm/min for the notched tension samples (R = 5, 0.1 mm), which gives an approximate strain rate on the order of 10–3/s for all the tests.

**Figure 4:**
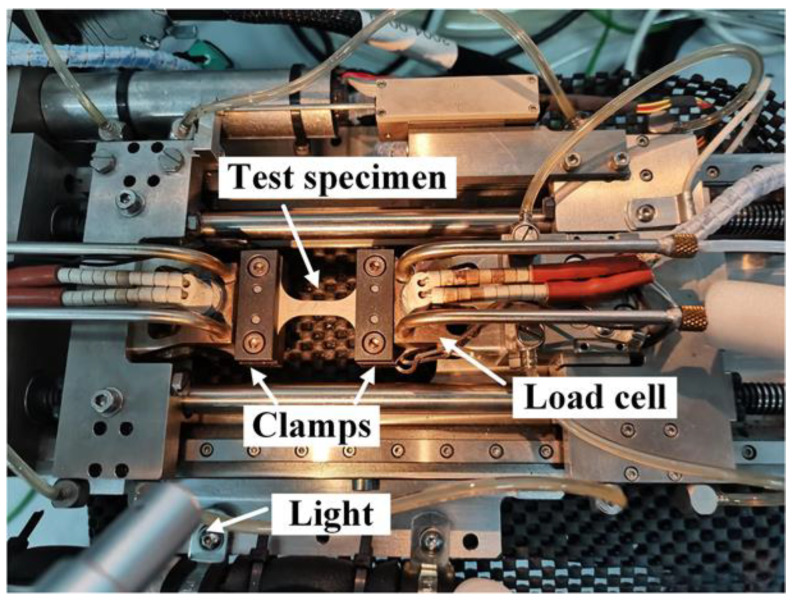
DIC system for tensile test.

All the uniaxial and notched tension tests were observed by a 2D-DIC system, shown in Figure 4. For that, before each test the front surface of the sample was cleaned with sandpaper and acetone, and white paints were sprayed first on the surface as the background, and then black paints were randomly sprayed to make a stochastic pattern. A UNIQ (Model UP-1830) CCD camera (UNIQ vision, Inc., Santa Clara, CA, USA) with a resolution of 1024 × 1024 pixels was utilized to capture continuous images at a constant rate of 1 Hz. The ratio of the physical size and the image pixel was 0.03 mm/pixel. The subset size of 33 pixels was chosen and the step size was 2 pixels. The surface displacements and strains were calculated by the VIC-2D software (Correlated Solutions Inc., Columbia, SC, USA). The relative displacements along the axial direction are obtained by virtual DIC extensometers with a gage length of 10 mm for all the specimens, the measuring points are the yellow dots shown in Figure 2. Moreover, through scanning with an electron microscope (Gemini SEM 300, Carl Zeiss, Oberkochen, Germany), the fracture surfaces of the tested samples were used to analyze the fracture characteristics.

## 3. Results

### 3.1. Loading Curves

**Figure 5:**
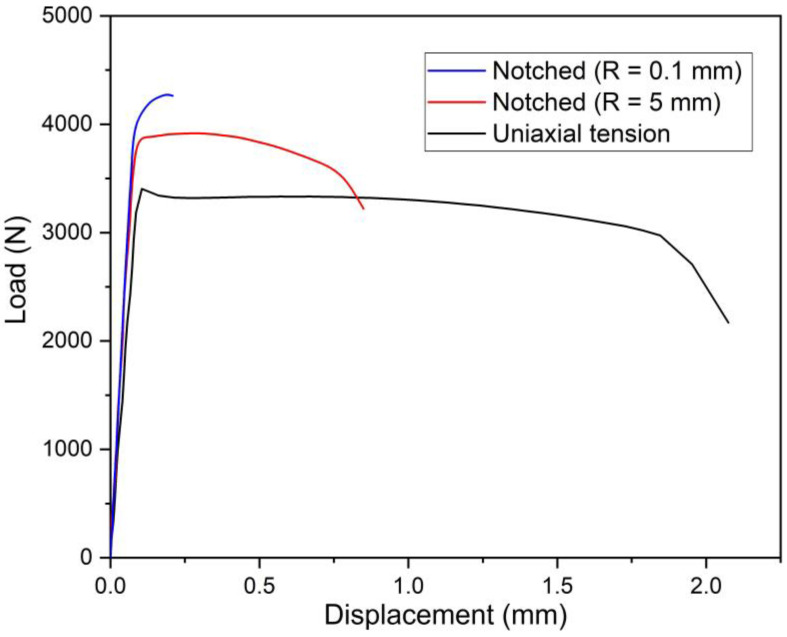
Load—displacement curves for the uniaxial tensile sample and the notched samples with a notch radius of 5 mm and 0.1 mm respectively.

The representative load—displacement curves for the uniaxial and notched tension specimen of Ti6Al4V are presented in Figure 5. It can be seen that the slope of the uniaxial test is slightly different from the notched samples. Because less material was removed from the notched geometry, the stiffness of the notched tensile samples is higher, which was similarly observed by Yang et al. [33]. The peak forces are 3414N, 3916N, and 4278N for the uniaxial tensile, and the notched specimens (R = 5 and 0.1 mm), respectively. The displacement at failure decreases significantly with the decrease of radius of the notch.

### 3.2. Uniaxial Tensile Test

**Figure 6:**
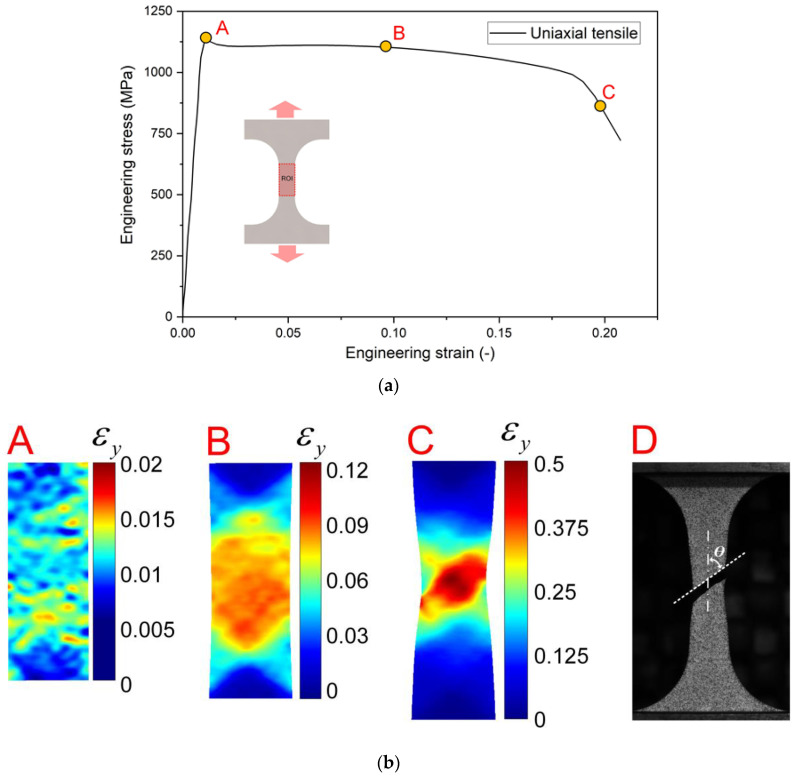
Loading curve and strain maps of uniaxial tension specimen: (**a**) Engineering stress-strain curve; (**b**) strain fields $\varepsilon_{y}$ at different loading points A, B, C indicated in (**a**) and D is the image of fractured specimen after test.

The stress—strain curve and strain evolution for the uniaxial tension specimen is presented in Figure 6. The engineering stress is calculated by dividing the measured force by the cross section of the specimen, while the engineering strain is obtained by the initial distance and relative displacement of the two yellow points in Figure 2a by the initial distance. At the initial loading stage, the stress increases linearly until it approaches a maximum of 1134 MPa, and the magnitudes of strains $\varepsilon_{y}$ on the specimen surface are very small at loading point A in Figure 6b(A). The strain $\varepsilon_{y}$ distributes homogeneously in the gage section when the engineering strain is about 0.1 (Figure 6b(B)). In the subsequent loading, the strain begins to localize in the central area of the sample, and it appears as a cross-shaped band just before the fracture. The crack initiates in the localized region and propagates along the localized band. A slant fracture is exhibited in the tensile sample. The angle θ of the fracture surface with respect to the loading axis is about 55° as shown in Figure 6b, which indicates that the initiation and propagation of crack is affected by the shear stress.

**Figure 7:**
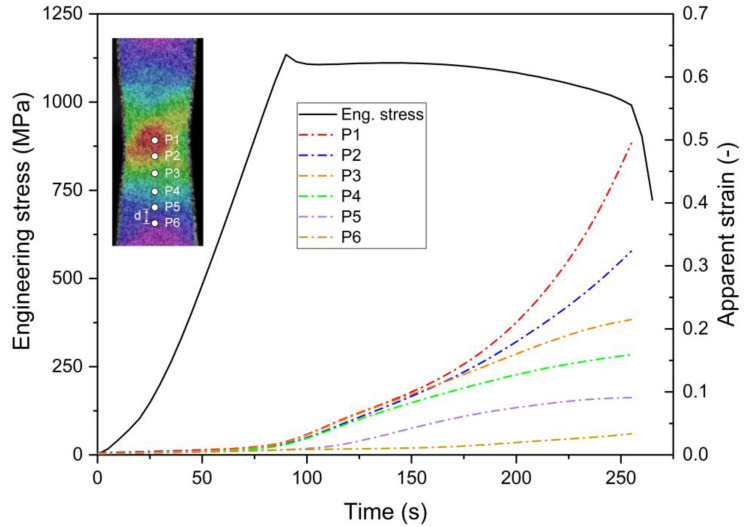
Loading curve of uniaxial tensile test and the evolution of the apparent strain at different positions (P1–P6) on the sample surface.

Figure 6 shows clearly the localized necking behavior of Ti6Al4V. Here, six points on the specimen surface were chosen to show the evolution of strain $\varepsilon_{y}$ with loading. Point 1 was selected in the center of the localized band region, whereas other points are selected at a distance of d, as shown in Figure 7. The distance d is 1 mm in the present study. The strain $\varepsilon_{y}$ at points P1 to P6 is represented on the secondary vertical axis of the graphs. At the initial stage of loading, the magnitudes of strains of all the selected points increase very slowly. Beyond loading point B (t = 160 s), the magnitudes of strains of P1 and P2 increase rapidly. Meanwhile, the increments of the strains at P3–P6 are slow at this stage, as shown in Figure 7. Therefore, points P1 and P2 correspond to the localized region on the specimen, and obviously points P3–P6 are not in the localized zone. The width of the localized zone is estimated to be approximately 2 mm. It can be seen that the strain localization usually occurs before the specimen fracture; the crack which initiates in the localized zone leads to the sudden failure of the material.

### 3.3. Notched Specimen Tests

**Figure 8:**
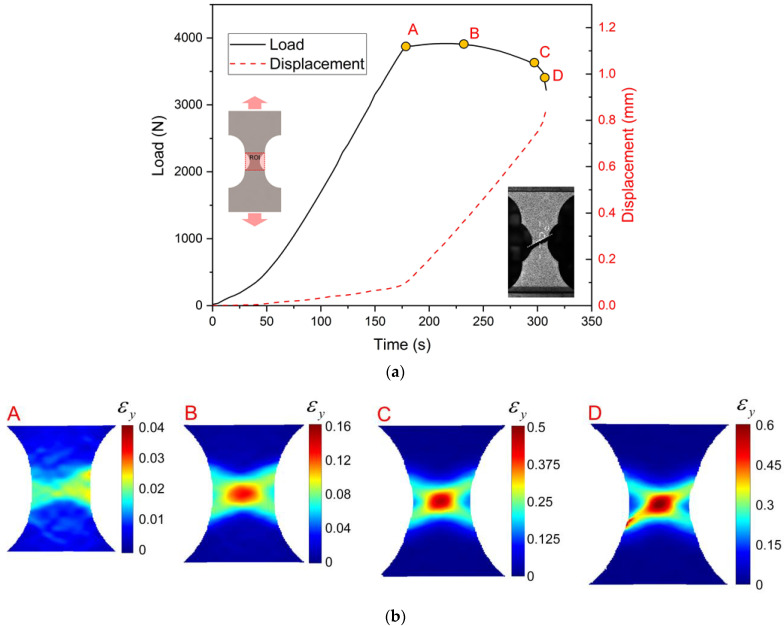
Loading curves and the strain fields in the notched tension sample (R = 5 mm) test: (**a**) Load—time and displacement—time curves; (**b**) apparent strain fields $\varepsilon_{y}$ at different loading stages.

Figure 8 exhibits the load—time curve and the apparent strain $\varepsilon_{y}$ fields for the notched tension sample (R = 5 mm). During the initial stage of loading, the force increases rapidly and the relative displacement of the two gage points increases almost linearly. Figure 8b(A) indicates that the magnitude of strain $\varepsilon_{y}$ is still small at loading point A. Then the load increases slowly to the maximum of 3916N and the strains start to localize in the central region of the sample shown in Figure 8b. The strain localization continues to develop and it evolves into two cross-shaped belts (Figure 8b(C,D)), which similarly appear in the uniaxial tensile tests. The fracture occurs soon with the increment of displacement in the localized necking region. The fracture surface is aligned with the localized neck, which is about θ = 60° to the loading direction (Figure 8b). This shows that the shear stress also has a significant influence on the crack initiation of the notched specimen (R = 5 mm).

**Figure 9:**
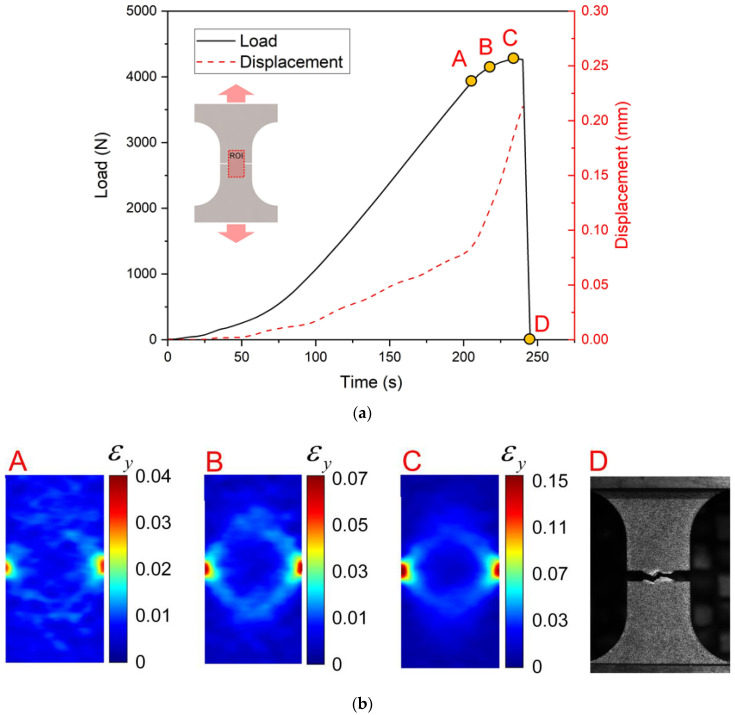
The loading curves and apparent strain maps for the notched tension sample (R = 0.1 mm): (**a**) Load—time and displacement—time curves; (**b**) the apparent strain $\varepsilon_{y}$ fields at loading points A–C and D specimen after failure.

The load—time curves and the apparent strain maps in the notched tension sample (R = 0.1 mm) are presented in Figure 9. It is noticed that the strains mainly localize around two notches, and that the strains in other regions are relatively small, as shown in Figure 9b(A). Beyond loading point A, the displacement started to grow rapidly, as the strains concentrated further at the notch tips shown in Figure 9b(B,C). At point C in 230 s seconds, the load increases to the maximum of 4278N. The load drops suddenly after the peak load. The fracture is initiated near the notches of the sample, and the maximum magnitude of apparent strains $\varepsilon_{y}$ are about 0.15, which are much lower compared to the uniaxial tension sample. The strain localization induces the crack nucleation and the propagation, which lead to the ultimate failure of the sample as shown in Figure 9b(D).

## 4. Discussion

### 4.1. Strain Hardening Behavior

**Figure 10:**
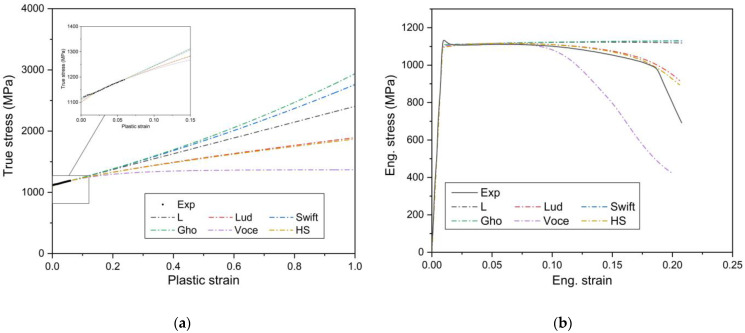
Comparison of simulation and experimental results of uniaxial tension test for: (**a**) True stress-plastic strain curves of the identified hardening laws; (**b**) engineering stress—strain curves.

The hardening laws often used in engineering practice are given in Table 1. σ and $\varepsilon_{p}$ represent the true stress and the plastic strain, and C1, C2, and C4 are the material constants which are needed to be identified. The least squares method (LSM) is used to fit between each of the hardening models and the experimental data in the pre-necking regime of the uniaxial tensile tests. The corresponding parameters for the data fitting are given in Table 2, and fitting results are displayed in Figure 10. At the pre-necking stage, fairly good agreement between the fitted and the measured curves is achieved using different models, and the difference of different hardening models is noticeable as the plastic strain increases. The Voce model underestimates the engineering stress—strain curve while other models overestimate (Figure 10b). It can be seen that the accuracy of the hardening models, which is utilized to predict the engineering stress—strain curve, is very sensitive to the extrapolated stress at a large strain.

$\sigma =C_{1}+C_{2}\cdot \varepsilon_{p}^{}$ $\sigma =C_{1}+C_{2}\cdot \varepsilon_{p}^{C_{3}}$ $\sigma =C_{1}\cdot {\left(C_{2}+\varepsilon_{p}\right)}^{C_{3}}$ $\sigma =C_{1}\cdot {\left(\varepsilon_{p}+C_{2}\right)}^{C_{3}}-C_{4}$ $\sigma =C_{1}+C_{2}\cdot \left[1-\exp(-C_{3}\cdot \varepsilon_{p})\right]$ $\sigma =C_{1}+C_{2}\cdot [1-\exp(-C_{3}\cdot \varepsilon_{p}{}^{C_{4}})]$

Figure 10a shows that the true stress of the linear hardening law increases proportionally, while the stress from the Swift law is above the experimentally obtained curve and the Voce curve is below the experimental stress. The stress from the Voce model tends to be constant after a certain extrapolation of stress because of the saturated expression of the Voce hardening law. Subsequently, these three descriptions are combined as a hybrid Linear—Swift—Voce (LSV) model which is expressed as

(10) $$\sigma =p_{1}\sigma_{L}+p_{2}\sigma_{S}+p_{3}\sigma_{V}$$

(11) $$p_{1}+p_{2}+p_{3}=1$$

where $\sigma_{L}$, $\sigma_{S}$, $\sigma_{V}$ are the stresses of the Linear, Swift and Voce laws in Equations (4), (6) and (8). Three weight parameters $p_{1}$, $p_{2}$ and $p_{3}$ vary between 0 and 1, while the sum of these parameters is 1.

**Figure 11:**
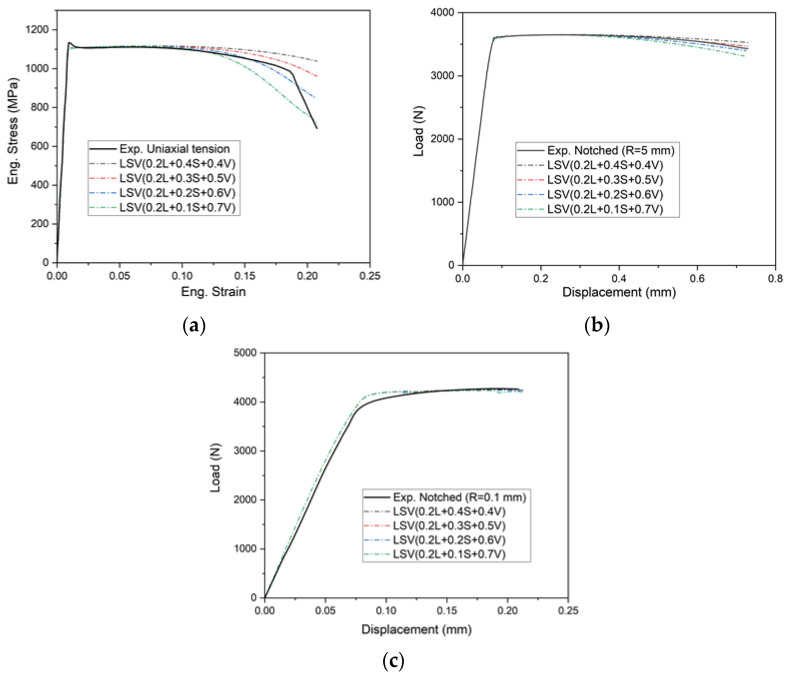
Comparison of loading curves from numerical and experimental results for: (**a**) uniaxial tension; (**b**,**c**) notched tension (R = 5, 0.1 mm) samples.

Simulations without the failure model are performed and the results are shown in Figure 11. The parameter $p_{1}$ is 0.2, $p_{2}$ changes from 0.1 to 0.4, and the sum of the three coefficients is equal to 1. The numerical results are inconsistent with the experiments when $p_{2}$ is about 0.2 or 0.3 in Figure 11a. The predicted load—displacement curve in the notched (R = 5 mm) specimen also indicates that $p_{2}$ is between 0.2 and 0.3 in Figure 11b. Figure 11c displays that the simulation curves of the notched tension test (R = 0.1 mm) are a little higher than the experimental results.

### 4.2. Influence of Stress Triaxiality on the Fracture Characteristics

**Figure 12:**
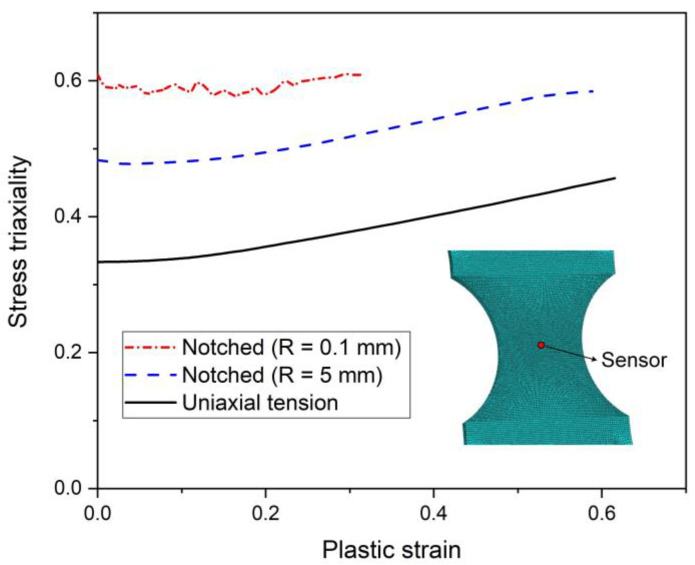
The evolution of stress triaxiality η with the plastic strain at the location showing in the inset.

The experimental results in Section 3 clearly show that the fracture characteristics strongly are affected by the loading conditions. Figure 12 presents the variation of the stress triaxiality η at the center of the sample with the plastic strain in the uniaxial and the notched tension tests. The LSV hardening model is used to perform the simulations. The experimental results in Section 3 clearly show that the fracture characteristics strongly are affected by the loading conditions. The stress triaxialities of the uniaxial tensile and the notched (R = 5 mm) specimens increase with the increase in the plastic strain. For the notched (R = 0.1 mm) tension sample, the stress triaxiality remains about 0.6 as the plastic strain increases.

**Figure 13:**
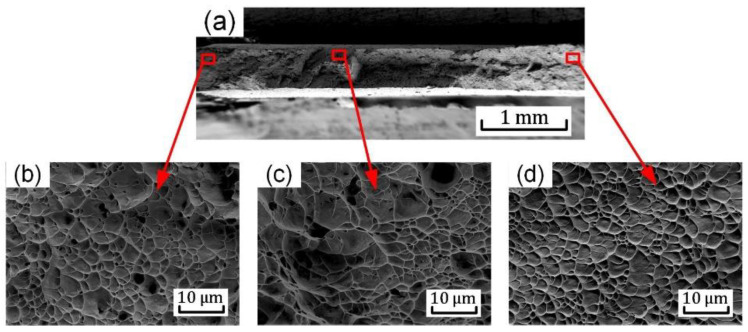
SEM fractography for uniaxial tension sample: (**a**) overall view of the fracture surface; (**b**–**d**) images at different locations of the fracture surface shown in (**a**).

After the mechanical tests, the fracture surfaces of different samples were extensively investigated. The SEM images of the fracture surface of uniaxial tension sample are shown in Figure 13. A large number of dimples were observed at the central area, which exhibit obvious features of ductile fracture (Figure 13c). Deep and small dimples are observed at the central area, which indicate the formation and growth of voids, inducing the crack initiation at the center of the sample. The average size of the dimples is about 2.5 μm. The dimples are elongated in a particular orientation near the edge regions of the fracture surface (Figure 13b,d); these are often termed shear dimples to indicate the influence of shear effect on the border of the specimen. The slant fracture occurs in the uniaxial tension tests shown in Figure 6b(D). The fracture of the uniaxial tension sample is induced by the combination of tensile and shear stress.

**Figure 14:**
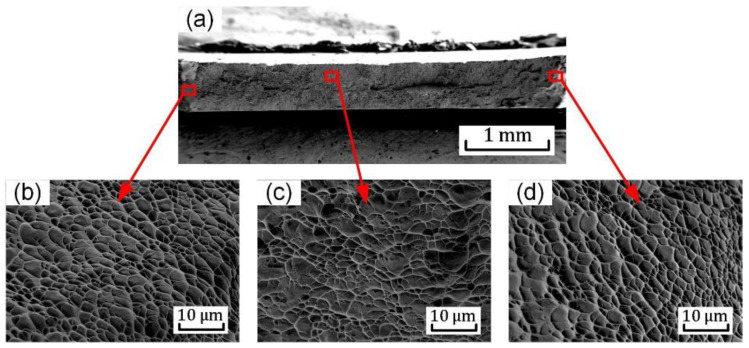
SEM images of feracture surface for the notched (R = 5 mm) tension sample: (**a**) overall view; (**b**–**d**) high magnification images of the regions highlighted in (**a**).

The fracture surface of the notched (R = 5 mm) tension sample is presented in Figure 14a. In comparison with the uniaxial tension sample, the dimples are larger and shallower (Figure 14c), as the average size of the dimple is about 4 μm. The shear dimples are also observed at the edge region of the notched sample (R = 5 mm) in Figure 14b. The strains localize as two crossed bands at the central area (Figure 8b(C)) and the inclined fracture in the notched specimen shows that shear stress strongly affects the damage and failure of Ti6Al4V.

**Figure 15:**
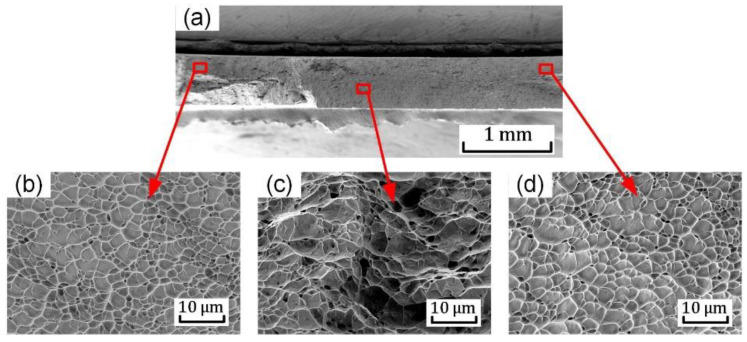
SEM images of fracture surface for the notched (R = 0.1 mm) tension sample: (**a**) overall view; (**b**–**d**) images of the regions shown in (**a**).

Figure 15 displays the fracture surface of the notched (R = 0.1 mm) tension specimen as the stress triaxiality at the notches is about 0.63. Shallow and equiaxed dimples are present near the two notches of the specimen shown in Figure 15b,d. The average dimple size is approximately 5 μm. From the distribution of the apparent strain on the notched (R = 0.1 mm) tension sample in Figure 9, the strains are highly concentrated near the two notches of the specimen. This shows that the higher stress triaxiality promotes the formation, growth, and coalescence of voids, leading to the crack initiation at the two notched regions. The propagation of the crack induces the final fracture of the sample. Comparing the fracture surfaces of the different samples, it can be inferred that as the stress triaxiality increases from 0.33 to 0.58, the average size of the dimples increases but the depth decreases.

**Figure 16:**
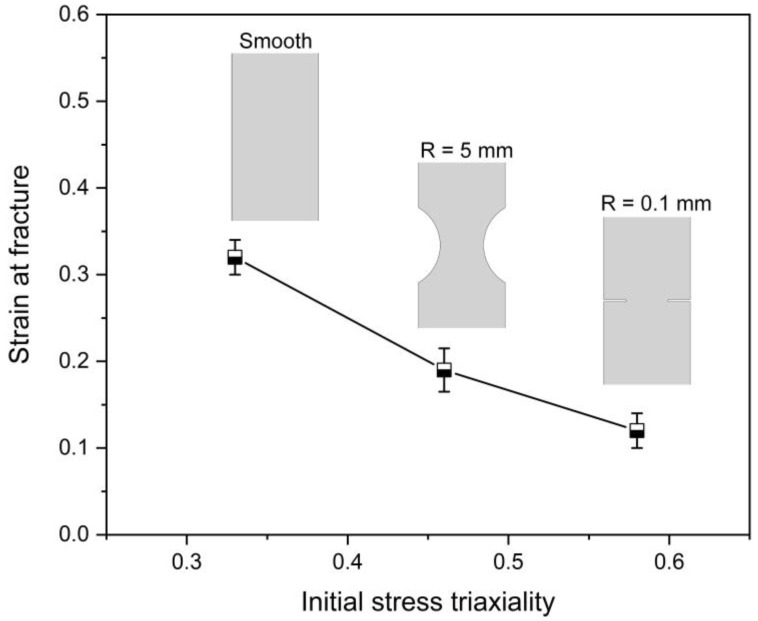
Strain at fracture sensitivity to the sample geometry.

The strain at fracture can be calculated by the equation $\varepsilon_{f}=\ln(A_{0}/A_{f})$, in which $A_{0}$ is the area of initial cross-section and $A_{f}$ is the area of fracture surface after failure. To determine the geometry of $A_{f}$, the width and inclined fracture angle can be obtained from the image of sample just before failure. The profile and thickness of the sample fracture surface can be acquired from the SEM images. Thus, the strains at fracture of different specimens can be obtained by the combined use of DIC measurement and SEM analysis, the results of which given in Figure 16. The fracture strain is about 0.32 when the initial stress triaxiality η=0.33, and the strain at failure decreases as the initial stress triaxiality η increases.

## 5. Conclusions

In this work, the strain hardening and failure of Ti6Al4V uniaxial and notched tension samples were investigated. DIC method was utilized to measure the strain fields of each sample during the loading. The experimental results indicated that the localization of strain usually occurs before fracture, the crack initiates in the localized zone and the propagation of the crack induces the final failure. The strains in the region of the strain localization increased rapidly after necking, and this characterized the width and the size of the localized zone. Slant fractures were observed in the uniaxial and the notched tension specimens, which indicated that the initiation and the propagation of the crack in the thin sheet specimen were highly affected by the shear stress.

Finite element simulations were performed for the uniaxial and notched tension specimens. A hybrid hardening model of Linear—Swift—Voce (LSV) was proposed to represent the strain hardening behaviors of Ti6Al4V. The parameters of the hardening law were identified and the numerical results were consisted with the experiments.

By analyzing the microstructure of the fracture surface, the void formation and growth were significantly affected by the plastic strain and the stress states. The dimples in the notched specimens were larger and shallower than that in uniaxial specimens. With the increment of the stress triaxiality, such as from the smooth to the notched tension samples, the ductility of these specimens decreases significantly. At a higher stress triaxiality, Ti6Al4V showed a damage mechanism of the micro void formation, growth, and coalescence, while at a low stress triaxiality, fracture developed as a combination of the void growth and the shear modes, which were supported by SEM observation of the fracture surfaces.

## Figures and Tables

**Table 1:** Various hardening laws.

Hardening Law	Abbreviation	Expression	Equation	Type
Linear	L	$\sigma =C_{1}+C_{2}\cdot \varepsilon_{p}^{}$	(4)	Linear
Ludwik (1909) [34]	Lud	$\sigma =C_{1}+C_{2}\cdot \varepsilon_{p}^{C_{3}}$	(5)	Power law
Swift (1952) [35]	S	$\sigma =C_{1}\cdot {\left(C_{2}+\varepsilon_{p}\right)}^{C_{3}}$	(6)	Power law
Ghosh (1977) [36]	Gho	$\sigma =C_{1}\cdot {\left(\varepsilon_{p}+C_{2}\right)}^{C_{3}}-C_{4}$	(7)	Power law
Voce (1948) [37]	V	$\sigma =C_{1}+C_{2}\cdot \left[1-\exp(-C_{3}\cdot \varepsilon_{p})\right]$	(8)	Saturation
Hockett-Sherby (1975) [38]	HS	$\sigma =C_{1}+C_{2}\cdot [1-\exp(-C_{3}\cdot \varepsilon_{p}{}^{C_{4}})]$	(9)	Saturation

**Table 2:** Calibration results of hardening models.

Hardening Model	Variables			
	C1	C2	C3	C4
L	1114.505	1290.356	-	-
Lud	1101.109	792.622	0.774	-
Swift	301.709	1.859	2.107	-
Gho	19.209	3.165	3.525	0.223
Voce	1108.564	259.898	6.333	-
HS	1100.329	32,851.668	0.024	0.765

## Data Availability

All the data is available within the manuscript.
